# A Hierarchical Voting Based Mixed Filter Localization Method for Wireless Sensor Network in Mixed LOS/NLOS Environments

**DOI:** 10.3390/s18072348

**Published:** 2018-07-19

**Authors:** Yan Wang, Jinquan Hang, Long Cheng, Chen Li, Xin Song

**Affiliations:** 1 Department of Computer and Communication Engineering, Northeastern University, Qinhuangdao 066004, China; hangjinquan@126.com (J.H.); 15028575121@163.com (C.L.); sxin78916@neuq.edu.cn (X.S.); 2 School of Information Science and Engineering, Northeastern University, Shenyang 110819, China

**Keywords:** wireless sensor network, non-line of sight, mobile localization, square root unscented Kalman filter, particle filter, convex optimization

## Abstract

In recent years, the rapid development of microelectronics, wireless communications, and electro-mechanical systems has occurred. The wireless sensor network (WSN) has been widely used in many applications. The localization of a mobile node is one of the key technologies for WSN. Among the factors that would affect the accuracy of mobile localization, non-line of sight (NLOS) propagation caused by a complicated environment plays a vital role. In this paper, we present a hierarchical voting based mixed filter (HVMF) localization method for a mobile node in a mixed line of sight (LOS) and NLOS environment. We firstly propose a condition detection and distance correction algorithm based on hierarchical voting. Then, a mixed square root unscented Kalman filter (SRUKF) and a particle filter (PF) are used to filter the larger measurement error. Finally, the filtered results are subjected to convex optimization and the maximum likelihood estimation to estimate the position of the mobile node. The proposed method does not require prior information about the statistical properties of the NLOS errors and operates in a 2D scenario. It can be applied to time of arrival (TOA), time difference of arrival (TDOA), received signal (RSS), and other measurement methods. The simulation results show that the HVMF algorithm can efficiently reduce the effect of NLOS errors and can achieve higher localization accuracy than the Kalman filter and PF. The proposed algorithm is robust to the NLOS errors.

## 1. Introduction

The wireless sensor network (WSN) is a network that consists of hundreds of tiny sensor nodes. The sensor nodes are randomly deployed in the monitoring field and they work cooperatively to gather physical information through wireless links [1]. The measured data is sent to a Fusion Center [2], which either uses the data locally or delivers the data to clients and servers [3]. WSN have been used in various applications, such as event detection (fires, floods) [4], monitoring (health care, industrial, agricultural, environmental) [5,6], energy-efficient routing [7,8], exploration (underground and undersea) [9], and surveillance [10].

Global positioning system (GPS) is one of the well-known solutions to the outdoor positioning problem, but the localization accuracy of GPS cannot achieve the requirements of indoor positioning [11]. The WSN based indoor localization has attracted much attention and become a research hotspot in recent years [12]. There are two types of nodes in the WSN based localization system: beacon and mobile node. The node with known coordinate information is called the beacon node, while the node without a prior coordinate is defined as the mobile node. The mobile node measures the distance [13] or angle from the beacon nodes, then it estimates the position of itself through a generic localization algorithm [14]. The developed localization algorithm should be able to provide a tradeoff between accuracy, robustness, and complexity. Indoor localization systems based on WSN technologies [15,16,17,18,19] have been used in a variety of applications, such as monitoring workers and valuable equipment in buildings, tracking products and transportation vehicles in logistics, and localizing prisoners in a jail [18].

For the localization methods based on WSN, the four main measurement methods to locate the mobile node consist of received signal strength (RSS) [20], angle of arrival (AOA) [21], time of arrival (TOA) [22] or time difference of arrival (TDOA) [23]. If there is direct propagation, also known as line-of-sight (LOS), between the beacon nodes and the mobile node, we can obtain the accurate position of the mobile node through the filtering algorithms. However, one of the major challenges in wireless positioning technology is the non-line of sight (NLOS) problem [24], which occurs when direct line-of-sight is blocked between the beacon and mobile nodes. In the case of NLOS, the propagation time of the signal is increased because the radio waves are reflected by the scatter or penetrate the blocking object [25]. Therefore, the WSN based localization in the complex NLOS environment is still a challenging problem.

In this paper, we propose a hierarchical voting based mixed filter (HVMF) localization algorithm to mitigate the NLOS error, which is suitable for a 2D scenario. We firstly use the hierarchical voting method to obtain the initial position estimation of a mobile node, and the probability of including the NLOS errors is obtained. Then, a mixed square root unscented Kalman filter (SRUKF) and particle filter (PF) method based on the probability is proposed to filter the larger measurement error. Finally, the convex optimization and maximum likelihood estimation method is proposed to estimate the position of the mobile node. The main contributions of this paper are given as follows:

(1) The proposed condition detection and distance correction method based on hierarchical voting does not require identification of the propagation state, and it is independent of the physical measurement ways.

(2) A mixed SRUKF and PF method is proposed to filter the larger measurement error. The proposed method only needs the parameter of measurement noise in the LOS condition. It does not require any prior information about the NLOS errors. Therefore, the proposed method can be widely used in other wireless localization methods.

(3) In this paper, we assume that the measurement model is based on TOA. The proposed localization method could easily extend to other measurement model such as TDOA and RSS.

(4) We compare the performance of the proposed method when the NLOS error obeys different distributions. The simulation results show that the proposed method is robust to the NLOS errors.

This paper is organized as follows. In Section 2 we elaborate on the related works. In Section 3, the problem statement and a brief introduction to SRUKF and PF are introduced. In Section 4 our proposed method is described in detail. Section 5 shows the simulation results. The conclusions are given in Section 6. The list of key notations is shown in Table 1. 

## 2. Related Works

The methods of combining all LOS and NLOS measurements to compute the position of mobile nodes have been studied in [26,27,28,29,30,31]. These methods do not require NLOS identification because they either use adaptive methods to adjust the probabilities of each model, or transform the positioning problem into sub-problems and eliminate NLOS errors through sub-problems. In [26,27,28,29], the interacting multiple model (IMM) approach and the data fusion algorithm [27,28] are investigated to mitigate the NLOS error. Based on the IMM approach, the switched model sets based interacting multiple model (SMS-IMM) algorithm [29] has been proposed. It takes the advantage of the switching between different model sets for further performance improvement. A selective fuzzy-tuned extended Kalman filtering based IMM (SFT-EKF-IMM) algorithm has been put forward in [27]. It presents a viable Bayesian estimation alternative to mobile localization enhancement and relies on a synergistic combination of valid aggregate measurements, NLOS bias modeling and estimation, and computational intelligence. In the IMM approach, two state space model sets are proposed. The model set 1 only considers the dynamics of a mobile station without covering the NLOS bias variation. The model set 2 consists of the modeling of the MS dynamics and the NLOS bias variation expressed as a random walk process. The two models apply to the LOS and NLOS environments, respectively. However, most of the above mentioned methods assume that the distribution or parameters of the NLOS error is known, which is impractical. In [30,31], an estimator is proposed by transforming the localization problem into a generalized trust region sub-problem framework. The proposed estimator is strictly decreasing over a readily obtained interval, and thus, can be solved exactly by a bisection procedure. The new approach does not require any assumptions about the statistics of NLOS bias, nor distinguish which link is NLOS or LOS [31]. In [32], an improved residual weighting (Rwgh) algorithm is proposed, which uses different subgroups of range measurements to eliminate the NLOS error. The algorithm does not require any prior information about the statistical properties of NLOS errors.

In [33,34,35,36,37,38,39], the NLOS identification algorithms have been proposed to realize mobile localization in NLOS environments. In [33,34,35], the NLOS measurements are identified and discarded in location estimation. In order to identify the propagation conditions (LOS or NLOS), the NP theorem is applied in [33] to determine a threshold value to the AOA. In [34], an improved method based on the Rwgh algorithm is proposed to gradually eliminate the NLOS transmission situation, then only the LOS measurements are used to localization. This method can be used in other measurement methods. Moreover, Bayes sequential test method [35] can also identify the propagation condition. To mitigate the impact of the NLOS propagation, the NLOS measurements are corrected by subtracting the mean of NLOS errors. The proposed method owns relatively higher localization accuracy in compare with other methods. In [36,37,38], methods of reconstructing NLOS measurement values have been proposed. Rather than processing all measurements via a single filter, the proposed algorithm in [36] distributes the measurements among several local filters. Through the distributed filtering and data association techniques, abnormal measurements due to NLOS are identified, and negative effects can be prevented. Besides, the hybrid particle finite impulse response filter (HPFF) was used to process localization failures due to NLOS. In [37], the modified Kalman filter algorithm is used to reduce the NLOS error according to its distribution model. And the least square method (LSM) method and the reconstructed measured value are combined to estimate the location of the target node. In [38], two algorithms based on machine learning and a third based on hypothesis testing have been proposed to separate the LOS/NLOS measurements. The key technology of the proposed method is exploiting several statistical features of the RSS time series, which are shown to be particularly effective. In [39], the data association method is proposed to mitigate the NLOS measurements. In this method, the binary hypothesis is carried out to detect the measurements that contain the NLOS errors. For the NLOS propagation condition, a mean shift algorithm is utilized to evaluate the means of the NLOS measurements and the data association method is used to alleviate the NLOS errors. It can be proved that the proposed method can provide higher location accuracy in comparison with some traditional methods. In our previous paper [40], we propose a RSS based localization algorithm in a NLOS environment. An algorithm for identifying NLOS errors using loop iteration is firstly proposed. Then we correct the NLOS measurements by subtracting the mean of NLOS errors. Finally, the Kalman filter is employed to mitigate the process noise. The previous paper [40] only applies to RSS, while this paper applies to a variety of situations that can convert measurement results into distance, such as TOA, TDOA, RSS, and other measurement methods. At the same time, the previous algorithm needs to know the mean of NLOS errors in advance, while this paper does not need the prior knowledge of NLOS errors.

## 3. Problem Statement

### 3.1. Signal Model

In this section, we introduce some technical preparation. We assume that *N* beacon nodes ($Z_{i}={\left[x_{i},y_{i}\right]}^{T},i=1,\ldots ,N$) are randomly deployed in the field. The location of the obstacles is unknown. The mobile node moves randomly in the field, at time *k* the position of mobile node is $Z_{u}^{t}={\left[x_{k}^{u},y_{k}^{u}\right]}^{T},k=1,\ldots ,t_{u}$. This paper considers a 2-D localization scenario.

The beacon node sends a signal; the mobile node receives the signal and converts it into distance information. In LOS propagation conditions, the estimation of TOA is modeled as follows:

(1) $${\hat{t}}_{TOA}=t_{TOA}+n_{TOA}$$

where $t_{TOA}$ is the true TOA between the beacon node and mobile node, $n_{TOA}$ is the measurement noise modeled as zero mean white Gaussian with variance $\sigma^{2}$. 

In LOS propagation conditions, the measured distance of the *i*-th beacon node at time *k* is modeled as [41]:

(2) $${\hat{d}}_{k}^{i}=c\cdot {\hat{t}}_{TOA}=d_{k}^{i}+n_{i}$$

where, $n_{i}$ is the measurement noise modeled as zero mean white Gaussian with variance $\sigma_{i}^{2}$, $d_{k}^{i}=\sqrt{{\left(x_{k}^{u}-x_{i}\right)}^{2}+{\left(y_{k}^{u}-y_{i}\right)}^{2}}$ is the true distance between the *i*-th beacon node and the mobile node at time *k*.

In the condition of NLOS propagation, due to the presence of obstacles, the signal transmission to the mobile node does not travel in a straight line. It will result in a reflection or diffusion effect. Therefore, the measured distance measurement of the *i*-th beacon node at time *k* is modeled as [42]:

(3) $${\hat{d}}_{k}^{i}=d_{k}^{i}+n_{i}+b_{NLOS}$$

where $n_{i}$ is the measurement noise with zero mean and $\sigma_{i}^{2}$ variance, i.e., $N\left(0,\sigma_{i}^{2}\right)$. $b_{NLOS}$ is the NLOS error and assumes that it is independent with the measurement noise $n_{i}$. Since the indirect propagation path is longer than the direct path, the NLOS error is assumed positive. And the NLOS error $b_{NLOS}$ obeys Gaussian, uniform, or exponential distribution. The distribution and parameters of $b_{NLOS}$ are different in different indoor environments and measurement methods. 

The probability density function of $n_{i}$ can be described by

(4) $$f\left(n_{i}\right)=\frac{1}{\sqrt{2\pi \sigma_{i}^{2}}}\exp\left(-\frac{n_{i}^{2}}{2\sigma_{i}^{2}}\right)$$

The probability density function of $b_{NLOS}$ when it obeys the Gaussian distribution ($b_{NLOS}\sim N\left(\mu_{NLOS},\sigma_{NLOS}^{2}\right)$) is given by

(5) $$f\left(b_{NLOS}\right)=\frac{1}{\sqrt{2\pi \sigma_{NLOS}^{2}}}\exp\left(-\frac{{\left(b_{NLOS}-\mu_{NLOS}\right)}^{2}}{2\sigma_{NLOS}^{2}}\right)$$

The probability density function of $b_{NLOS}$ when it obeys the Uniform distribution ($b_{NLOS}\sim U\left(u_{\min},u_{\max}\right)$) is given by

(6) $$f\left(b_{NLOS}\right)=\{\begin{array}{cc} \frac{1}{u_{\max}-u_{\min}} & ,for\;u_{\min}\leq b_{NLOS}\leq u_{\max}\\ 0 & ,else \end{array}$$

The probability density function of $b_{NLOS}$ when it obeys the Exponential distribution ($b_{NLOS}\sim \exp\left(\lambda \right)$) is given by

(7) $$f\left(b_{NLOS}\right)=\{\begin{array}{cc} \lambda^{-1}e^{-b_{NLOS}/\lambda } & ,b_{NLOS}\geq 0\\ 0 & ,b_{NLOS}<0 \end{array}$$

### 3.2. A Brief Introduction to SRUKF and PF

UKF (unscented Kalman filter) is a method of approximating nonlinear distribution using sampling strategy. It is based on UT (Unscented Transformation) transformation and adopts a Kalman linear filtering framework. The specific sampling form is deterministic sampling. Research results show that UKF has the same computational complexity as the EKF algorithm, but its performance is better than that of EKF. It does not require the calculation of the Jacobian matrix and can approximate any posterior probability mean and covariance to second-order accuracy for any nonlinearity. It can avoid the sampling particle decay problem and other advantages.

The SRUKF (square root unscented Kalman filter) is based on the UKF and introduces the matrix QR decomposition, Cholesky factorization update, and least-squares linear algebra optimization to reduce the overhead of the algorithm. The essence of these techniques is based on the weighted statistics linear regression technique, using a priori distribution to construct a set of deterministic sampling points (Sigma points) to capture the relevant statistical parameters of the system, and use linear regression to transform the Sigma points to represent the state’s posterior distribution. This statistical linearization technique takes full account of the statistical properties of Gaussian random variables.

The PF (Particle filtering) is the most representative non-linear filtering implementation method in the Bayesian recursive framework. It uses a set of particles with weights (sample points) obtained through random sampling to fit the target after the state space distribution. The probability density function is tested to replace the integral operation with the sample mean to obtain the state minimum variance distribution. When the sample size is large enough, it can approximate any form of probability density distribution. The advantages of the PF are also reflected in its complexity and precision only in relation to the number of particles and the PF algorithm itself, independent of the dimension of the state space. In this way, the PF will not cause performance degradation or increase in complexity due to the increase in the dimension of the state space. Although the probability distribution in the algorithm is only an approximation of the real distribution, due to the non-parametric characteristics, it can get rid of the constraints that the random variables must satisfy the Gaussian distribution when solving the nonlinear filtering problem. It can express a wider distribution than the Gaussian model. It also has stronger modeling capabilities for the nonlinear characteristics of variable parameters. Therefore, the PF can more accurately express the posterior probability distribution based on the observation and control quantities. 

## 4. Proposed Method

**Figure 1:**
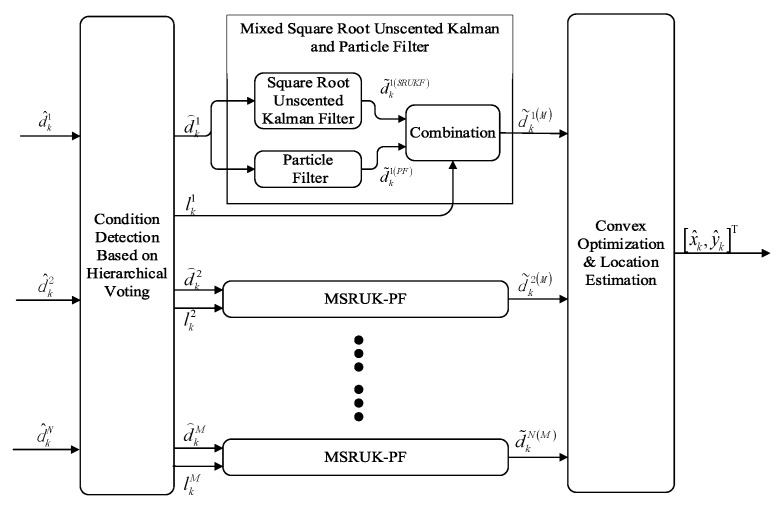
The flowchart for the proposed algorithm.

As shown in Figure 1, the input of the method is the measurement distance ${\hat{d}}_{k}^{i}$ and the output of the method is position of mobile node ${\left[{\hat{x}}_{k},{\hat{y}}_{k}\right]}^{T}$, the method consists of three main stages: 

(**1**). **Condition Detection and Distance Correction based on Hierarchical Voting:**

We estimate the initial position of the mobile node by voting based on the obtained data ${\hat{d}}_{k}^{i}$. Then we calculate the corrected distance ${\overset{⌢}{d}}_{k}^{i}$ of each node based on the initial position, and give the probability $l_{k}^{i}$ of the measurement contains NLOS error.

(**2**). **Mixed Square Root Unscented Kalman and Particle Filter**

Because SRUKF is mainly applied to nonlinear Gaussian distributions, PF is mainly applied to nonlinear non-Gaussian distributions. Therefore, we decided to combine the two algorithms to get better results. First we should perform SRUKF and PF using on the corrected distance ${\overset{⌢}{d}}_{k}^{i}$, and then mix the values of the two filters to obtain the mixed measurement value ${\tilde{d}}_{k}^{i\left(M\right)}$ using the probability $l_{k}^{i}$.

(**3**). **Convex Optimization & Location Estimation**

First, we perform convex optimization using the mixed measurement values ${\tilde{d}}_{k}^{i\left(M\right)}$ of each beacon node. Finally, the maximum likelihood estimation method is used to estimate the position of mobile node ${\left[{\hat{x}}_{k},{\hat{y}}_{k}\right]}^{T}$ using the output of the convex optimization.

### 4.1. General Concept

Let *i*-th node obtain the measured value at time *k* as ${\hat{d}}_{k}^{i}$. The state vector at time *k* is defined by

(8) $$s_{k}^{i}={\left[{\hat{d}}_{k}^{i},{\dot{\hat{d}}}_{k}^{i}\right]}^{T}$$

where *T* denotes the transpose operator, ${\hat{d}}_{k}^{i}$ is the velocity of the mobile node. And the variance of the system is defined as $P_{k}$, with $P_{k}=U_{k}{}^{T}U_{k}$.

Then the state equation of *i*-th beacon node under LOS/NLOS environment can be expressed as follows:

(9) $$s_{k}^{i}=F_{k}s_{k-1}^{i}+G_{k}\omega_{k-1}$$

where the state transition matrix $F_{k}$ is defined as $F_{k}=\left[\begin{array}{cc} 1 & \Delta t\\ 0 & 1 \end{array}\right]$, $\Delta t=t_{k}-t_{k-1}$ is the sampling period. The vector $\omega_{k}$ is a zero-mean white Gaussian noise process with diagonal covariance matrix $Q=\sigma_{i}^{2}I$. And the matrix $G_{k}$ is defined as $G_{k}={\left[\frac{\Delta t^{2}}{2},\Delta t\right]}^{T}$.

The measurement equation of *i*-th beacon node under LOS/NLOS environment can be expressed as follows:

(10) $$z_{k}^{i}=H_{k}s_{k}^{i}+v_{k}$$

where $H_{k}=\left[1,0\right]$, and $v_{k}$ can be written as:

(11) $$v_{k}=\{\begin{array}{cc} v_{k}\sim N\left(0,\sigma_{i}^{2}\right) & ,LOS\\ v_{k}\sim N\left(\mu_{nlos},\sigma_{i}^{2}+\sigma_{NLOS}^{2}\right) & ,NLOS \end{array}$$

### 4.2. ConditionDetection and Distance Correction Based on Hierarchical Voting

In the condition detection and distance correction algorithm, the voting matrix is firstly established using the measurements. The initial estimated position is computed using the voting matrix which owns the largest value. Finally, the corrected distance and probability of the NLOS are estimated. We assume that the size of the field is *M* × *M*, and it is divided into *W* × *W* cells by integrating the estimation accuracy and computational complexity. The cell can be represented as C(m,n), for *m*, *n* = 1, …, *W*. The resolution of each cell is *w* (*w* = *M*/*W*). For example, a 100 × 100 field, if *W* = 10, the mesh resolution *w* is equal to 100/10 = 10.

At time *k*, the proposed algorithm includes the following steps:

***Step* 1:** We construct a *W* × *W* voting matrix *V*. The elements of the voting matrix are obtained in the following way:

(12) $$V\left(m,n\right)=\sum_{i=1}^{N}b_{i}\left(m,n\right),\;\begin{array}{cc} \mathrm{for} & m,n=1,\ldots ,W \end{array}$$

where $b_{i}\left(m,n\right)$ can be written as:

(13) $$b_{i}\left(m,n\right)=\{\begin{array}{c} X\left(d_{imn}-{\hat{d}}_{k}^{i}\right),\\ 0, \end{array}\begin{array}{c} X\left(d_{imn}-{\hat{d}}_{k}^{i}\right)\geq X\left(0\right)/2\\ X\left(d_{imn}-{\hat{d}}_{k}^{i}\right)<X\left(0\right)/2 \end{array}$$

where $d_{imn}$ is the Euclidean distance between *i*-th beacon node and the C(m,n), ${\hat{d}}_{k}^{i}$ is the measured distance of *i*-th node at time *k*. We define that $e_{k}^{i}\left(m,n\right)=d_{imn}-{\hat{d}}_{k}^{i}$, $X\left(e_{k}^{i}\left(m,n\right)\right)=N\left(e_{k}^{i}\left(m,n\right);0,\sigma_{i}^{2}\right)$ and $N\left(e_{k}^{i}\left(m,n\right);0,\sigma_{i}^{2}\right)$ denotes the Gaussian density function of $e_{k}^{i}\left(m,n\right)$ with zero mean and covariance $\sigma_{i}^{2}$.

***Step* 2:** We can obtain all the elements in the voting matrix V that have the largest value. They marked as $V\left(m^{*},n^{*}\right)$ and meet $V\left(m^{*},n^{*}\right)\geq V\left(m,n\right)$, for m,n=1,…,W. The initial estimated location of mobile node is $C^{*}=\left[C_{1}^{*},\ldots ,C_{v}^{*}\right]$. $C^{*}$ is the initial results set. And we can get the initial estimated position of the mobile node:

(14) $${\bar{C}}^{*}=\sum_{i}^{v}C_{i}^{*}/v$$

***Step* 3:** The corrected distance by *i*-th beacon node at time *k* is can be expressed as:

(15) $${\overset{⌢}{d}}_{k}^{i}=\left\|{\bar{C}}^{*}-Z_{i}\right\|$$

where $Z_{i}={\left[x_{i},y_{i}\right]}^{T}$ represents the location of *i*-th node.

***Step* 4:** The probability of the measurement contains NLOS error is

(16) lki=‖d^ki−d⌢ki‖∑i=1N‖d^ki−d⌢ki‖

**Figure 2:**
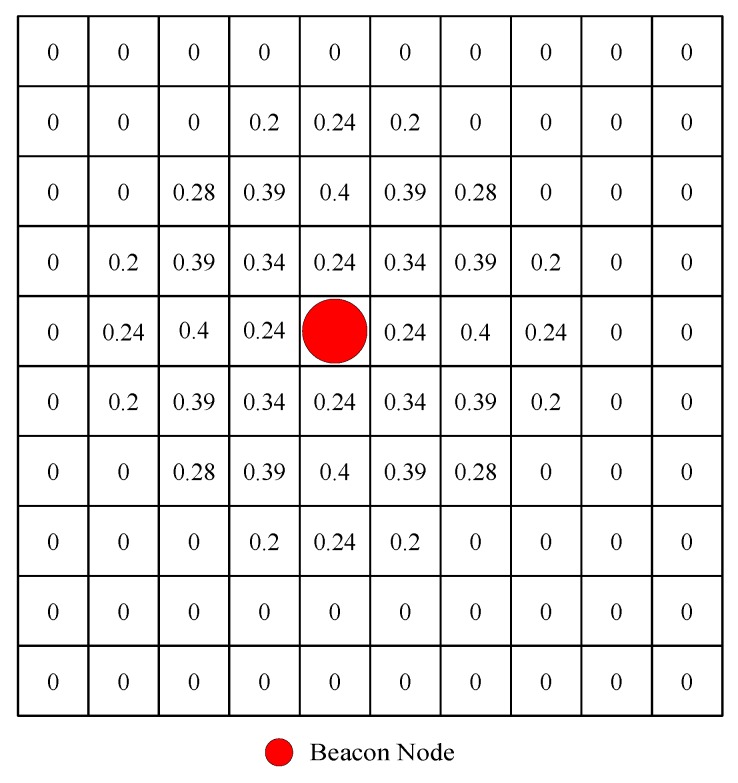
An example of voting process (only one beacon node).

To illustrate our voting process, we will provide two simple voting examples. Figure 2 shows a 100 × 100 field with *M* = 10, *w* = 10 and $\sigma_{i}^{2}$ = 1. The red dot indicates the location of the beacon node. The number on each grid represents the probability that the mobile node is at the grid when the distance measured by the beacon node from the mobile node is 2. 

**Figure 3:**
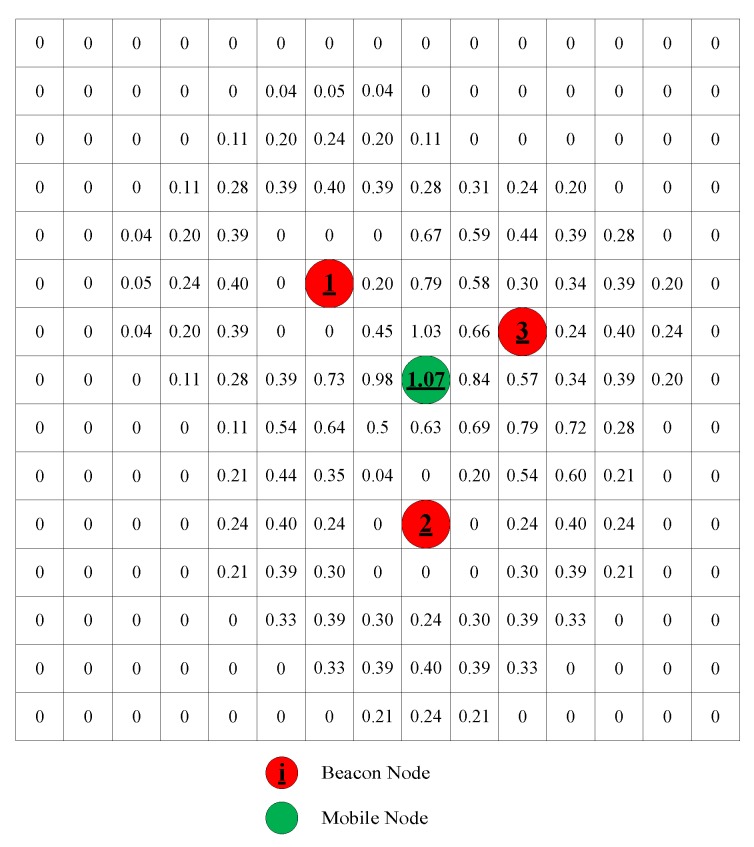
An example of voting process (three beacon node).

In Figure 2, it can be seen that the smaller the distance deviation is, the greater the probability. Figure 3 shows a 150 × 150 field with *M* = 15, *w* = 10 and $\sigma_{i}^{2}$ = 1. The red dot indicates the location of the beacon node. The green dot indicates the final estimated position. The distance measured by the beacon nodes from the mobile node is 2, 3, and 2 respectively. The number on each grid represents the probability that the mobile node is at the grid. It can be seen that the smaller the distance deviation is, the greater the probability.

Based on the aforementioned descriptions, the condition detection and distance correction based on hierarchical voting can be summarized as the pseudo code shown in Algorithm 1.

**Algorithm 1** Condition detection and distance correction based on hierarchical votingInput: ${\hat{d}}_{k}^{i}$ Output: $l_{k}^{i}$, ${\overset{⌢}{d}}_{k}^{i}$ Initialization: V=0 begin for *i* = 1:*N* do  for *m* = 1:*W* do   for *n*=1:*W* do    $d_{imn}=\left\|C\left(m,n\right)-Z_{i}\right\|$    $b_{i}\left(m,n\right)=\{\begin{array}{c} X\left(d_{imn}-{\hat{d}}_{k}^{i}\right),\\ 0, \end{array}\begin{array}{c} X\left(d_{imn}-{\hat{d}}_{k}^{i}\right)\geq X\left(0\right)/2\\ X\left(d_{imn}-{\hat{d}}_{k}^{i}\right)<X\left(0\right)/2 \end{array}$   end for end for   $V\left(m,n\right)=V\left(m,n\right)+b_{i}\left(m,n\right)$ end for $C_{i}^{*}=\left[m^{*},n^{*}\right]=find\left(V==\max\left(\max\left(V\right)\right)\right)$ ${\bar{C}}^{*}=\sum_{i}^{v}C_{i}^{*}/v$ for *i*=1:*N* do   ${\overset{⌢}{d}}_{k}^{i}=\left\|{\bar{C}}^{*}-Z_{i}\right\|$   lki=‖d^ki−d⌢ki‖∑i=1N‖d^ki−d⌢ki‖ end for end

### 4.3. Square Root Unscented Kalman Filter

The initial state vector is defined as $s_{0}^{i},i=1,\ldots ,M$. We can use the following formula to get the initial error variance $P_{0}$:

(17) $${\hat{s}}_{0}=E\left(s_{0}^{i}\right)$$

(18) $$P_{0}=E\left(\left(s_{0}^{i}-{\hat{s}}_{0}\right){\left(s_{0}^{i}-{\hat{s}}_{0}\right)}^{T}\right)$$

where $s_{k}^{i}={\left[{\overset{⌢}{d}}_{k}^{i},{\dot{\overset{⌢}{d}}}_{k}^{i}\right]}^{T}$.

Then we can compute a Cholesky factorization of $P_{0}$ to get its upper triangular factor $U_{i,0|0}=chol(P_{0}),i=1,\ldots ,M$.

Let $s_{i,k-1|k-1}$ be the estimated state and $U_{i,k-1|k-1}$ be the estimated error covariance matrix of the state, then the prior estimate of the state vector and the corresponding error covariance matrix can be obtained as:

(19) $$s_{i,k|k-1}=Fs_{i,k-1|k-1}$$

(20) $$U_{i,k|k-1}=qr\left\{\left[\begin{array}{c} U_{i,k-1|k-1}F^{T}\\ Q^{\frac{1}{2}}G^{T} \end{array}\right]\right\}$$

where the function qr{.} returns the upper triangular factor of the QR factorization of its matrix argument.

We can generate a set of estimated sigma points using $s_{i,k|k-1}$ and $U_{i,k|k-1}$:

(21) $$s_{i,k|k-1}^{\left(j\right)}=\{\begin{array}{l} s_{i,k|k-1},\\ s_{i,k|k-1}+\sqrt{\eta_{\alpha }}{\left(U_{i,k|k-1}^{T}\right)}_{j},\\ s_{i,k|k-1}-\sqrt{\eta_{\alpha }}{\left(U_{i,k|k-1}^{T}\right)}_{j-N_{v}}, \end{array}\begin{array}{l} j=0\\ j=1,\ldots ,N_{v}\\ j=N_{v}+1,\ldots ,2\cdot N_{v} \end{array}$$

where $N_{v}$ is the dimension of the state vector (in this paper, $N_{v}$ = 2), ${\left(U_{i,k|k-1}^{T}\right)}_{j}$ denotes the *i*-th column of matrix $U_{i,k|k-1}^{T}$, and $\eta_{\alpha }$ is a tuning parameter that controls the spread of the sigma points.

After that, we calculate the weight coefficient ω as follows:

(22) $$\omega^{\left(j\right)}=\{\begin{array}{ll} 1-\frac{N_{v}}{\eta_{\alpha }}, & j=0\\ \frac{1}{2\eta_{\alpha }}, & j=1,\ldots ,2\cdot N_{v} \end{array}$$

In the next step, we convert the vector $s_{i,k|k-1}^{\left(j\right)}$ obtained into a one-dimensional distance $z_{i,k|k-1}^{\left(j\right)}$ and use the weight coefficient to obtain the average distance ${\hat{z}}_{i,k|k-1}$:

(23) $$z_{i,k|k-1}^{\left(j\right)}=H_{k}s_{i,k|k-1}^{\left(j\right)},\;j=0,\ldots ,2\cdot N.$$

(24) $${\hat{z}}_{i,k|k-1}=\sum_{j=0}^{2N}\omega^{\left(j\right)}z_{i,k|k-1}^{\left(j\right)}$$

We calculate the value of the upper triangular Cholesky factor $U_{i,z|k}$ as follows

(25) $$e_{i,z}^{\left(j\right)}=\sqrt{\omega^{\left(j\right)}}\begin{array}{cc} \left(z_{i,k|k-1}^{\left(j\right)}-{\hat{z}}_{i,k|k-1}\right), & j=0,\ldots ,2\cdot N \end{array}$$

(26) $$U_{i,z|k}=qr\left\{{\left[e_{i,z}^{\left(0\right)},e_{i,z}^{\left(1\right)},\ldots ,e_{i,z}^{\left(2N\right)},R^{\frac{1}{2}}\right]}^{T}\right\}$$

The cross-covariance matrix $P_{i,k|k-1}^{s,z}$ is described as

(27) $$P_{i,k|k-1}^{s,z}=\sum_{j=0}^{2N}\omega^{\left(j\right)}\left(s_{i,k|k-1}^{\left(j\right)}-s_{i,k|k-1}\right){\left(z_{i,k|k-1}^{\left(j\right)}-{\hat{z}}_{i,k|k-1}\right)}^{T}$$

After completing the above steps, we calculate the filter gain matrix $T_{i,k}$ as follows:

(28) $$T_{i,k}=P_{i,k|k-1}^{s,z}U_{i,z|k}^{-1}\;$$

The posteriori state estimate $s_{i,k|k}$ and the Cholesky factor of the error covariance matrix $U_{i,k|k}$ can be updated as

(29) $$s_{i,k|k}=s_{i,k|k-1}+T_{i,k}U_{i,z|k}^{-T}\left({\overset{⌢}{d}}_{k}^{i}-{\hat{z}}_{i,k|k-1}\right)$$

(30) $$U_{i,k|k}=cholupdate\left\{U_{i,k|k-1},T_{i,k},-1\right\}$$

The filtered distance of SRUKF can be expressed as:

(31) $${\tilde{d}}_{k}^{i\left(SRUKF\right)}=H_{k}s_{i,k|k}$$

### 4.4. Particle Filter (PF)

We divide the PF step into four phases: initial phase, prediction phase, re-sampling phase, and output phase.

***Step* 1 (Initial Phase):**

We firstly initialize $s_{0}$, and randomly generate the particle swarm ${\left\{s_{0}^{n}\right\}}_{n=1}^{N_{s}}$ (where $N_{s}$ means the number of the particles, in this article $N_{s}=10$), and set the weight of all particles in the prior probability ${\left\{\omega_{0}^{n}\right\}}_{n=1}^{N_{s}}$ is $1/N_{s}$.

***Step* 2 (Prediction Phase):**

At the beginning of the filtering at each time *k*, the *i*-th particle and its weight at the previous time can be represented as $s_{i,k-1}^{n}$ and $\omega_{i,k-1}^{n}$. The prediction of the $s_{i,k}^{n}$ and the $\omega_{i,k}^{n}$ can be described by:

(32) $$s_{i,k}^{n}=s_{i,k-1}^{n}+N\left(0,\sigma_{i}^{2}\right),n=1,2,\ldots ,N_{s}$$

(33) $$z_{i,k}^{n}=H_{k}s_{i,k}^{n}$$

(34) $$\omega_{i,k}^{n}=\left(\frac{\sqrt{2\times \pi }}{\sigma_{i}}\times e^{-\frac{{\left(z_{i,k}^{n}-{\overset{⌢}{d}}_{k}^{i}\right)}^{2}\times \sigma_{i}^{2}}{2}}\right),n=1,\ldots ,N_{s}$$

After that we need to normalize the importance weights:

(35) $$\omega_{i,k}^{n}=\frac{\omega_{i,k}^{n}}{\sum_{\overset{⌢}{n}=1}^{N_{s}}\omega_{i,k}^{\overset{⌢}{n}}}$$

***Step* 3 (Re-sampling Phase):**

Generate two random number $N_{random}$ and $n_{random}$:

(36) $$N_{random}=2\times \max\left(\omega_{i,k}^{n}\right)\times rand\left(0,1\right)$$

(37) $$n_{random}=rand\left(0,1\right)$$

where the function rand(0,1) returns a pseudo random scalar drawn from the standard uniform distribution on the open interval (0,1).

Then do the following steps while $N_{random}>\omega_{i,k}^{n_{random}}$:

(38) $$N_{random}=N_{random}-\omega_{i,k}^{n_{random}}$$

(39) $$n_{random}=n_{random}+1$$

In the while loop if $n_{random}>N_{s}$, then $n_{random}=1$.

The estimated state vector for *i*-th particle is:

(40) $$s_{i,k}^{n}=s_{i,k}^{n_{random}}$$

***Step* 4 (Output Phase):**

The state estimate can be computed as:

(41) $${\hat{s}}_{i,k}=\sum_{n=1}^{N_{s}}\omega_{i,k}^{n}s_{i,k}^{n}$$

The filtered distance of PF can be expressed as:

(42) $${\tilde{d}}_{k}^{i\left(PF\right)}=H_{k}{\hat{s}}_{i,k|k}$$

### 4.5. Mixed Square Root Unscented Kalman and Particle Filter

In this subsection, we combine the results of SRUKF and PF. It can be expressed as:

(43) $${\tilde{d}}_{k}^{i\left(M\right)}=l_{k}^{i}\cdot {\tilde{d}}_{k}^{i\left(PF\right)}+\left(1-l_{k}^{i}\right){\tilde{d}}_{k}^{i\left(SRUKF\right)}$$

where $l_{k}^{i}$ is the probability that a measurement contains NLOS error according to Equation (16).

### 4.6. Convex Optimization & Location Estimation

Because the range of the NLOS error is too large, we optimize the result by using convex optimization. First, we generate a set of estimated sigma points by using the mixed measurement values ${\tilde{d}}_{k}^{i\left(M\right)}$.

(44) $$d_{k}^{\left(j\right)}=\{\begin{array}{l} {\tilde{d}}_{k}^{i\left(M\right)},\\ {\tilde{d}}_{k}^{i\left(M\right)}+\sqrt{\eta_{\alpha }}\sigma_{i},\\ {\tilde{d}}_{k}^{i\left(M\right)}-\sqrt{\eta_{\alpha }}\sigma_{i}, \end{array}\begin{array}{l} j=0\\ j=1\\ j=2 \end{array}$$

Perform convex optimization for the estimated sigma points, map the points that violate the constraints into the feasible region. The optimization scheme is as follows:

(45) $$\begin{array}{l} \underset{u}{\min}\left\{u\cdot u\right\}\\ s.t.\left\|\left(d_{k}^{\left(j\right)}-u\right)-{\tilde{d}}_{k}^{i\left(M\right)}\right\|\leq \sigma_{i},i\in N_{k} \end{array}$$

(46) $$P\left(d_{k}^{\left(j\right)}\right)=d_{k}^{\left(j\right)}-u$$

where $\sigma_{n}$ means the standard deviation of the measurement error, s.t. is the abbreviation of *subject to*, indicating that the latter formula is a constraint for convex optimization.

Using the $\omega^{\left(j\right)}$ and the optimized point $P\left(d_{k}^{\left(j\right)}\right)$, the final result is obtained by the following transformation:

(47) $${\tilde{d}}_{k}^{i}=\sum_{j=0}^{2}\omega^{\left(j\right)}P\left(d_{k}^{\left(j\right)}\right)$$

After that, we introduce the maximum likelihood localization method. We assume that the position of the beacon node is $\left(\left(x_{1},y_{1}\right),\ldots ,\left(x_{N},y_{N}\right)\right)$. At time *k*, the position of the mobile node ($Z_{u}^{t}={\left[x_{k}^{u},y_{k}^{u}\right]}^{T},k=1,\ldots ,t_{u}$) ${\tilde{d}}_{k}^{i}$ is the output of convex optimization. These values comply with the following formula:

(48) $$\{\begin{array}{c} {\left(x_{1}-x_{k}^{u}\right)}^{2}+{\left(y_{1}-y_{k}^{u}\right)}^{2}={\left({\tilde{d}}_{k}^{1}\right)}^{2}\\ \vdots \\ {\left(x_{N}-x_{k}^{u}\right)}^{2}+{\left(y_{N}-y_{k}^{u}\right)}^{2}={\left({\tilde{d}}_{k}^{N}\right)}^{2} \end{array}$$

It can be simplified as:

(49) $$\{\begin{array}{c} 2x_{k}\left(x_{1}-x_{2}\right)+2y_{k}\left(y_{1}-y_{2}\right)={\tilde{d}}_{k}^{2}-{\tilde{d}}_{k}^{1}-\left(x_{2}^{2}+y_{2}^{2}\right)+\left(x_{1}^{2}+y_{1}^{2}\right)\\ \vdots \\ 2x_{k}\left(x_{1}-x_{N}\right)+2y_{k}\left(y_{1}-y_{N}\right)={\tilde{d}}_{k}^{N}-{\tilde{d}}_{k}^{1}-\left(x_{N}^{2}+y_{N}^{2}\right)+\left(x_{1}^{2}+y_{1}^{2}\right) \end{array}$$

The above equation is represented by the linear equation AX=B, where A and B are given by:

$$A=2\left[\begin{array}{ccc} \left(x_{1}-x_{2}\right) & & \left(y_{1}-y_{2}\right)\\ \left(x_{1}-x_{3}\right) & & \left(y_{1}-y_{3}\right)\\ \vdots & & \vdots \\ \left(x_{1}-x_{N}\right) & & \left(y_{1}-y_{N}\right) \end{array}\right]B=\left[\begin{array}{c} {\tilde{d}}_{k}^{2}-{\tilde{d}}_{k}^{1}-\left(x_{2}^{2}+y_{2}^{2}\right)+\left(x_{1}^{2}+y_{1}^{2}\right)\\ {\tilde{d}}_{k}^{3}-{\tilde{d}}_{k}^{1}-\left(x_{3}^{2}+y_{3}^{2}\right)+\left(x_{1}^{2}+y_{1}^{2}\right)\\ \vdots \\ {\tilde{d}}_{k}^{N}-{\tilde{d}}_{k}^{1}-\left(x_{N}^{2}+y_{N}^{2}\right)+\left(x_{1}^{2}+y_{1}^{2}\right) \end{array}\right]$$

The final estimated position of the mobile node at time *k* is as follows:

(50) $${\left[{\hat{x}}_{k},{\hat{y}}_{k}\right]}^{T}={\left(A^{T}A\right)}^{-1}A^{T}B$$

## 5. Simulation and Experiment Results

### 5.1. Simulation Results

**Figure 4:**
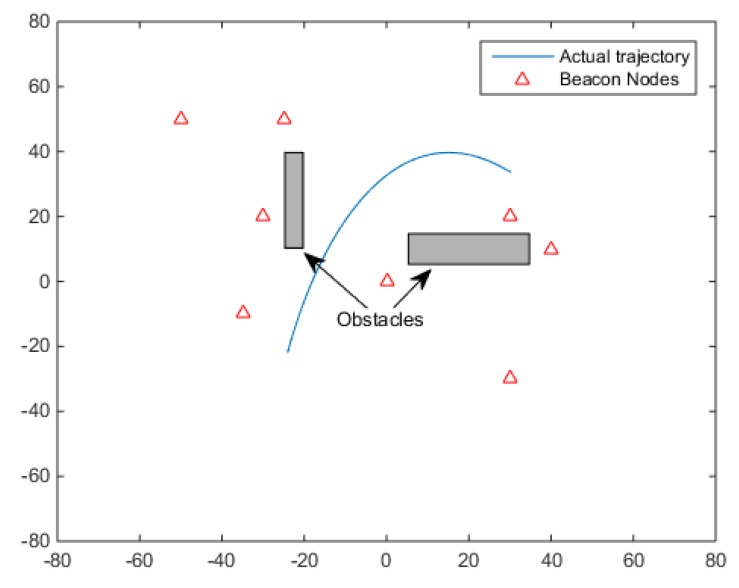
The deployment of beacon nodes and obstacles.

In this section, we present the simulation results for the proposed HVMF algorithm. As shown in Figure 4, we randomly deploy 8 beacon nodes in the 100 m × 100 m area, and one mobile node (MN) is moving in the area. We assume that the mobile node has the velocity of 1 m/s. The communication range of the sensor node is 150 m. The default parameter values in the simulation are shown in Table 2.

We compare the proposed method with no filter (NF) method, the Kalman filter (KF) method, and the Particle Filter (PF) algorithm. In each simulation case, 1000 Monte Carlo runs are performed with the same parameters. The performance of the proposed algorithm is measured by the Root Mean Square Error (RMSE):

(51) $$\mathrm{RMSE}=\sqrt{\frac{1}{K\cdot t_{n}}\sum_{i=1}^{t_{n}}\sum_{k=1}^{K}\left({\left(x\left(k\right)-{\hat{x}}_{i}\left(k\right)\right)}^{2}+{\left(y\left(k\right)-{\hat{y}}_{i}\left(k\right)\right)}^{2}\right)}$$

where *t_n_* = 1000, *K* = 91, [x(k),y(k)] is the true position of the mobile node at time *k*, and $\left[{\hat{x}}_{i}\left(k\right),{\hat{y}}_{i}\left(k\right)\right]$ is the estimated position for *i*-th trial at time *k*.

**Figure 5:**
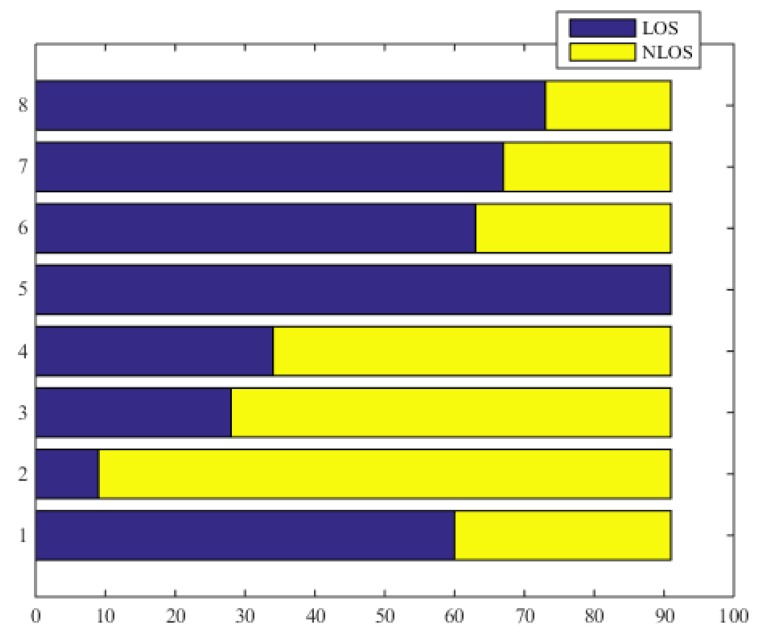
The sight state in sample points.

Figure 5 shows the sight state with respect to all the beacon nodes in the sample points. We can see that the sight states vary with time.

In the following section, we evaluation the performance of our proposed method under different environment; i.e., the NLOS errors obey different distribution. We also investigate the effect of various parameters on the proposed method.

#### 5.1.1. Large Measurement Noise

In this subsection, we consider the case of a narrow-band ranging application where the noise variance is relatively high, i.e., $\sigma_{i}$ = 3 is considered.

**Figure 6:**
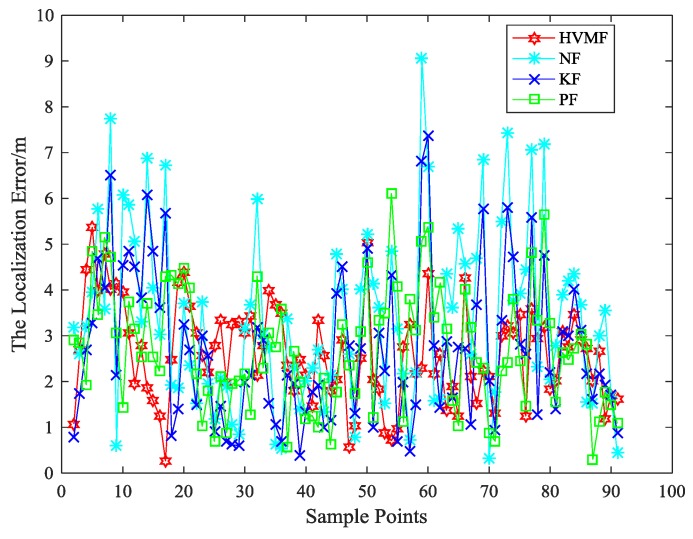
The localization error in each sample point.

When the number of beacon nodes is eight, the localization errors of four algorithms at each sample points are shown in Figure 6. As can be seen, the proposed method has better performance than the other methods in most sampling points.

**Figure 7:**
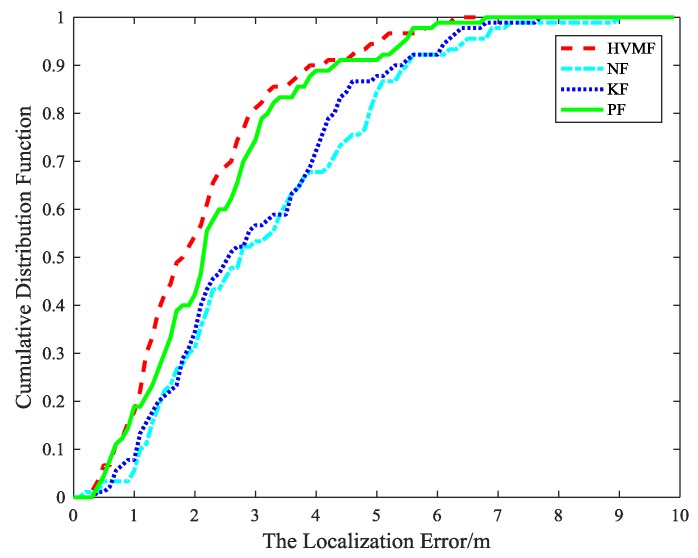
The localization error versus cumulative distribution function (CDF).

The cumulative distribution function (CDF) of the localization error is depicted in Figure 7. It can be observed that the 90-percentile average localization error of the HVMF algorithm is less than 4.0 m and the CDF tends to one at the localization error of less than 6.4 m. In contract, the 90-percentile average localization error of the KF, NF, and PF are achieved at 5.4 m, 5.6 m, and 4.4 m, respectively.

#### 5.1.2. Small Measurement Noise

For further verify our algorithm, we consider a case where the noise variance is relatively small; i.e., $\sigma_{i}$ = 1.

**Figure 8:**
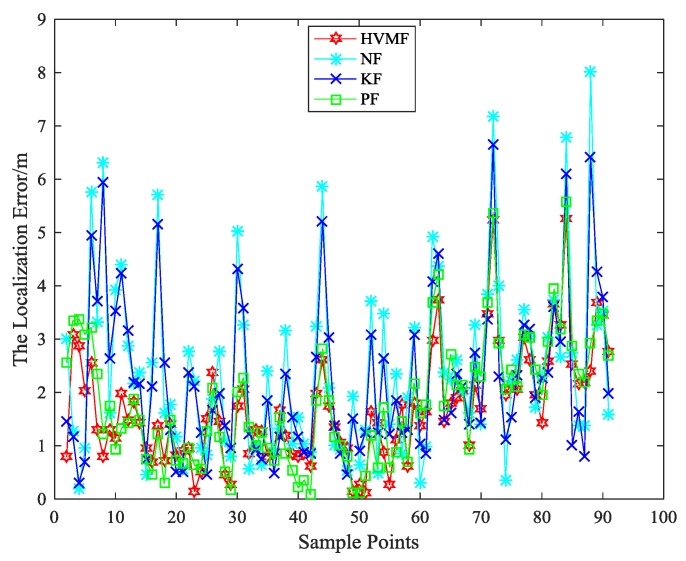
The localization error in each sample point.

**Figure 9:**
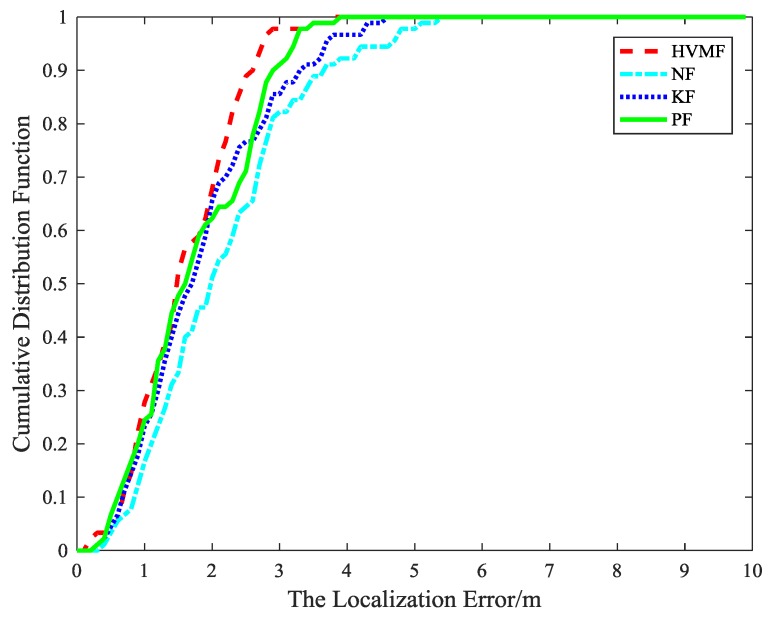
The localization error versus CDF.

The number of nodes is eight, the measurement errors of the four algorithms at each time are shown in Figure 8. As can be seen, the proposed method has better performance than the other methods in most sampling points. Figure 9 shows the cumulative distribution function of the localization error at each time. When the localization error is small, several algorithms have almost the same performance, but when the localization error is large, the HVMF algorithm has obvious advantages over other algorithms. The proposed method has more points within the average error 3 than NF, KF, and PF, about 20.9%, 13.04%, and 8.33%, respectively.

#### 5.1.3. The NLOS Errors Obey Gaussian Distribution

In this subsection, we assume that the NLOS error obey the Gaussian distribution; i.e., $b_{NLOS}\sim N\left(\mu_{NLOS},\sigma_{NLOS}^{2}\right)$.

**Figure 10:**
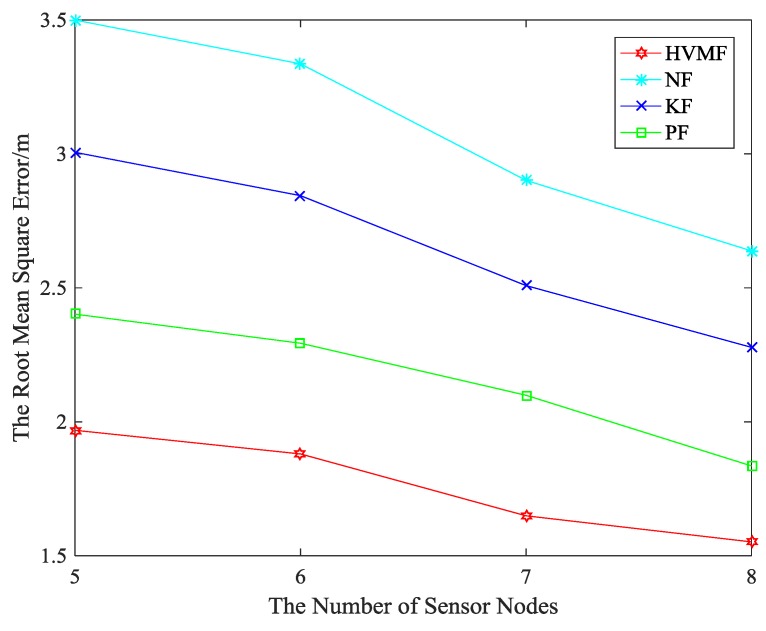
The number of beacon nodes versus Root Mean Square Error (RMSE).

The relationship between the RMSE and the number of beacon nodes is shown in Figure 10. Evidently, the proposed method also has the best positioning accuracy. The proposed method has higher localization accuracy than NF, KF, and PF, about 43.04%, 33.73% and 18.31%, respectively.

**Figure 11:**
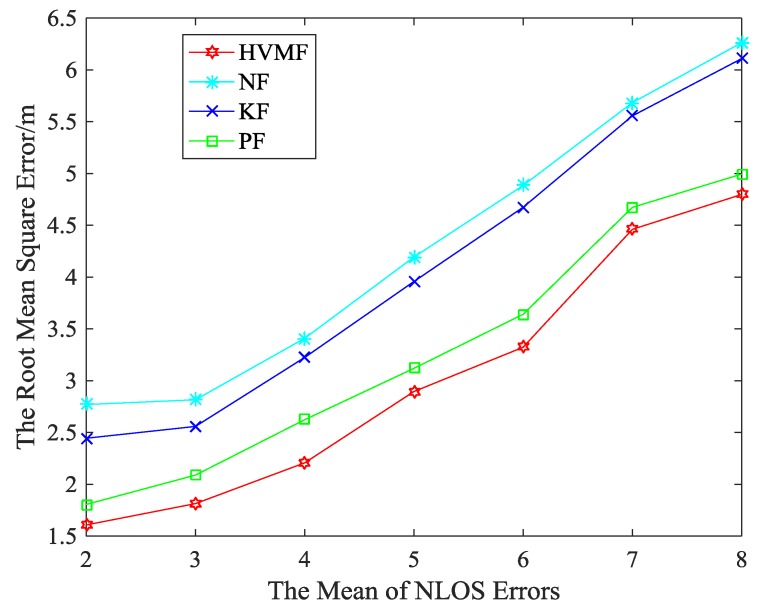
The mean of non-line of sight (NLOS) errors versus RMSE.

Figure 11 shows the effect of the mean of NLOS errors on the RMSE. We can see that the RMSE of all methods increases with the mean of NLOS errors increase. The proposed method has a higher localization accuracy than NF, KF, and PF, about 29.69%, 26.01%, and 8.02%, respectively.

**Figure 12:**
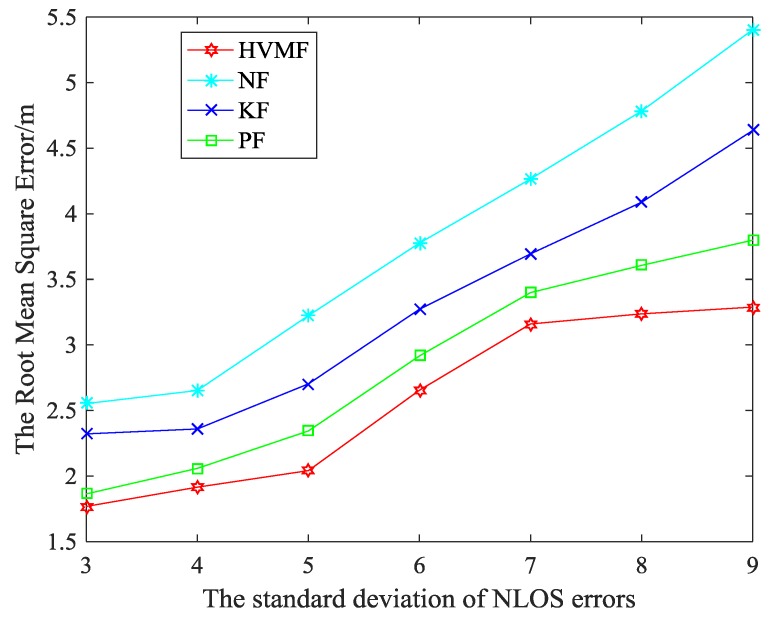
The standard deviation of NLOS errors versus RMSE.

Figure 12 shows the effect of the standard deviation of NLOS errors on the RMSE. We can see that the RMSE of all methods increases with the standard deviation of NLOS errors increase. At the same time, the HVMF algorithm has better performance when the deviation is large. When the standard deviation of NLOS errors is 3, the proposed method has higher localization accuracy than NF, KF, and PF, about 30.72%, 23.82%, and 5.11%, respectively. When the standard deviation of NLOS errors is 9, the proposed method has higher localization accuracy than NF, KF, and PF, about 39.14%, 29.05%, and 13.43%, respectively.

#### 5.1.4. The NLOS Errors Obey Uniform Distribution

In this subsection, we assume that the NLOS errors obey uniform distribution. The parameters of uniform distribution are 2 and $U_{\max}$, namely, $b_{NLOS}\sim U\left(2,U_{\max}\right)$.

**Figure 13:**
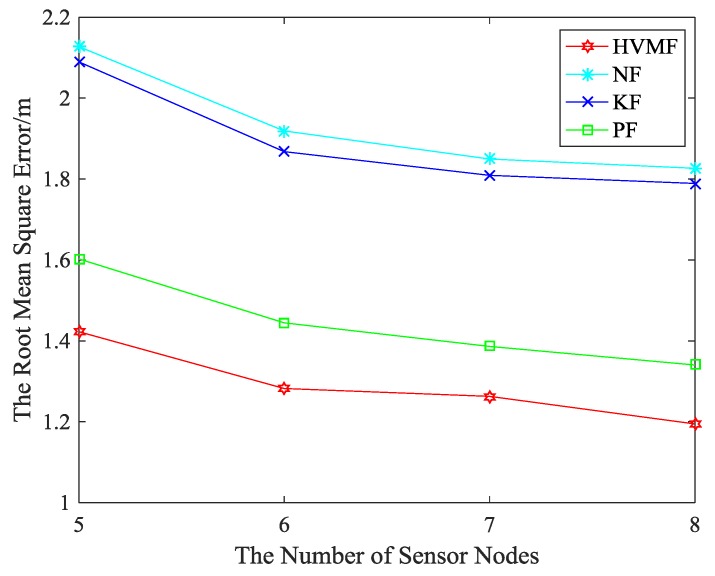
The number of beacon nodes versus RMSE.

Figure 13 shows the relationship between the RMSE and the number of beacon nodes. In Figure 13, the proposed method has higher localization accuracy than NF, KF and PF, about 33.15%, 31.67% and 10.59%, respectively.

**Figure 14:**
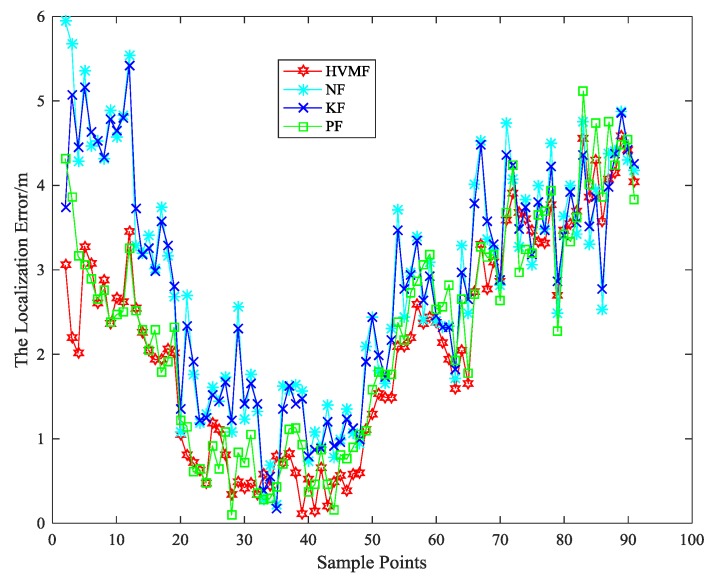
The localization error in each sample point.

When the number of nodes is eight, the localization error of each algorithm at each time is shown in Figure 14. As can be seen, the proposed method has better performance than the other methods in most sampling points.

**Figure 15:**
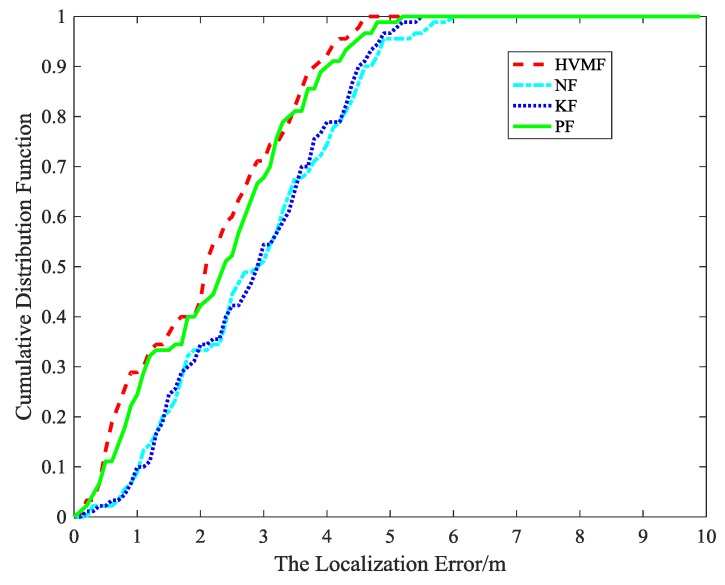
The localization error CDF.

The cumulative distribution function of the localization error is shown in Figure 15. It indicates that the 90-percentile localization error of the HVMF algorithm is less than 3.5 m and the CDF tends to one at the localization error of less than 4.7 m. However, the 90-percentile average localization error of the KF, NF, and PF are already arrived at 4.3 m, 4.4 m, and 3.7 m, respectively.

**Figure 16:**
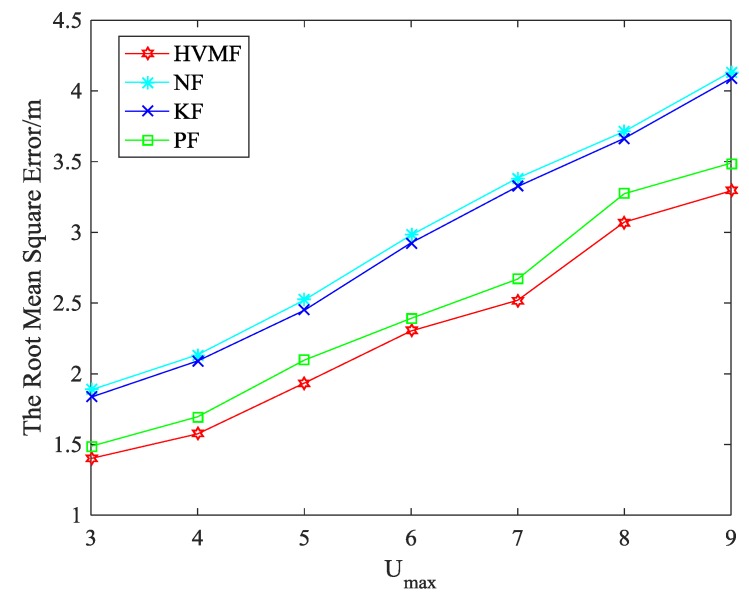
RMSE versus $U_{\max}$.

Figure 16 shows the effect of $U_{\max}$ on the RMSE when the NLOS error obeys uniform distribution, i.e., $b_{NLOS}\sim U\left(2,U_{\max}\right)$. We can see that the RMSE of all methods increases with the increase of $U_{\max}$. The proposed method has the highest localization accuracy than KF, PF and NF, and the advantage of the proposed method becomes more and more obvious.

#### 5.1.5. The NLOS Errors Obey Exponential Distribution

In this subsection, we evaluate the performance of the four methods when the NLOS errors obey exponential distribution, i.e., $b_{NLOS}\sim E\left(u\right)$.

**Figure 17:**
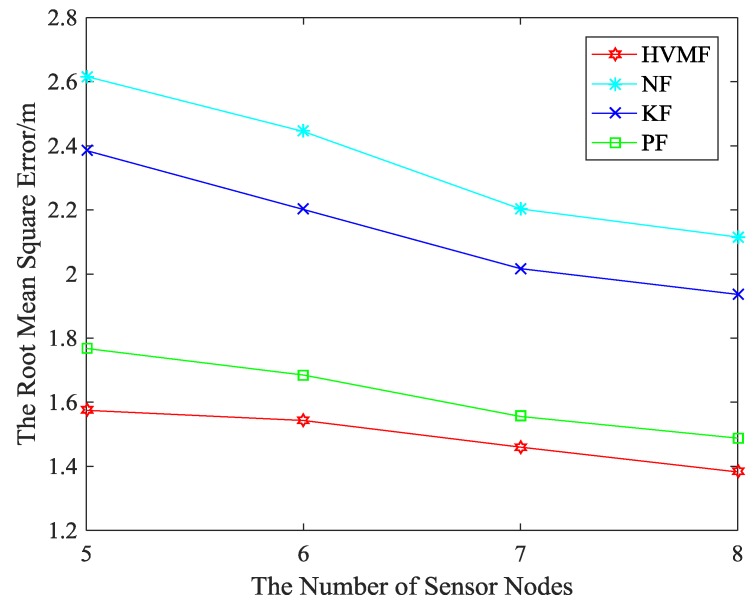
The number of beacon nodes versus RMSE.

The relationship between the RMSE and the number of beacon nodes can be seen in Figure 17. The proposed method has higher localization accuracy than NF, KF and PF, about 36.45%, 30.20%, and 8.25%, respectively.

**Figure 18:**
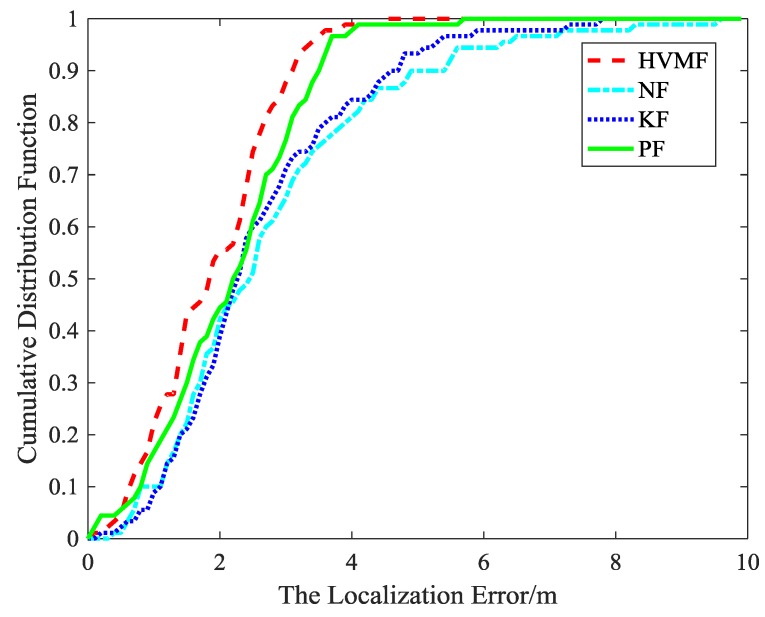
The localization error versus CDF.

Figure 18 is the cumulative distribution function of the localization error at each sample points. We can see that the advantage of the proposed method becomes more and more obvious. The proposed method has more points within the average error 3 m than NF, KF, and PF, about 24.73%, 19.57%, and 12.47%, respectively.

**Figure 19:**
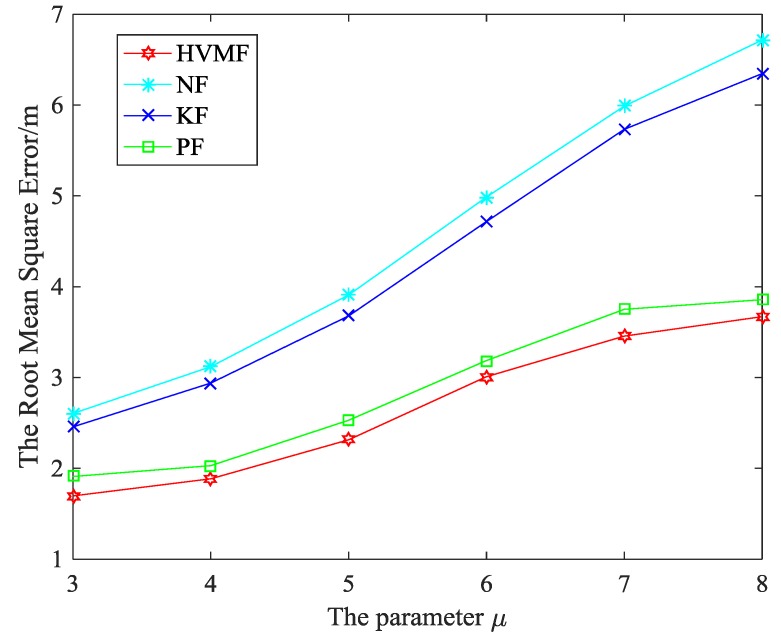
The parameter μ versus RMSE.

Figure 19 shows the performance of the proposed method when the NLOS error obeys the Exponential distribution. It can be observed that the RMSE increases with the parameter u increases. The proposed HVMF method has the highest localization accuracy, but the NF method owns the worst performance. At the same time, the HVMF and PF algorithm have better performance when the deviation is large. When the standard deviation of NLOS errors is three, the HVMF method has higher localization accuracy than NF, KF, and PF, about 34.85%, 30.95% and 11.20%, respectively. When the standard deviation of NLOS errors is 8, the HVMF method has higher localization accuracy than NF, KF, and PF, about 45.35%, 42.17%, and 4.85%, respectively.

### 5.2. Experiment Results

#### 5.2.1. Localization Results

**Figure 20:**
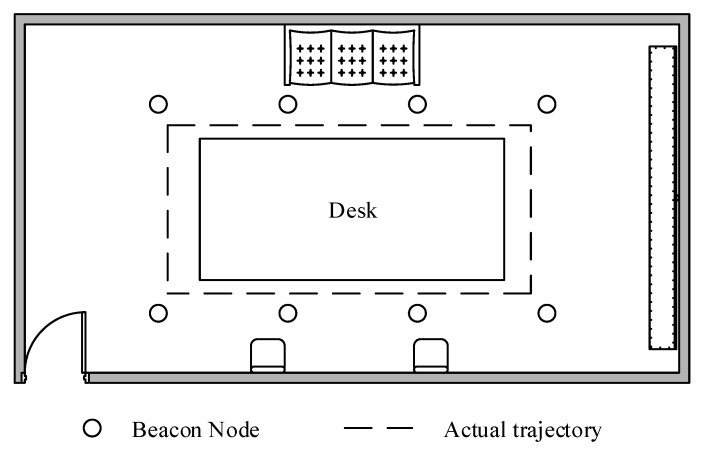
The floor plan for the test bed.

In order to test the localization performance of the proposed algorithm in a practical environment, we perform a realistic experiment in the building. The experimental equipment used for the experiment is a chirp spread spectrum (CSS) localization system, mainly consisting of CSS nodes. As shown in Figure 20, we deploy 8 beacon nodes in a 5 m × 7 m room. The beacon nodes and unknown nodes and are installed up to 1.7 m above the ground. MN moves around a rectangle table at a constant velocity. The distance measurement frequency of the CSS nodes is set to 20 Hz, and 20 distance measurements are carried out at each sampling site in order to weaken the negative impacts imposed to the localization accuracy. The average measurements at each sampling site are used for the localization calculation.

**Figure 21:**
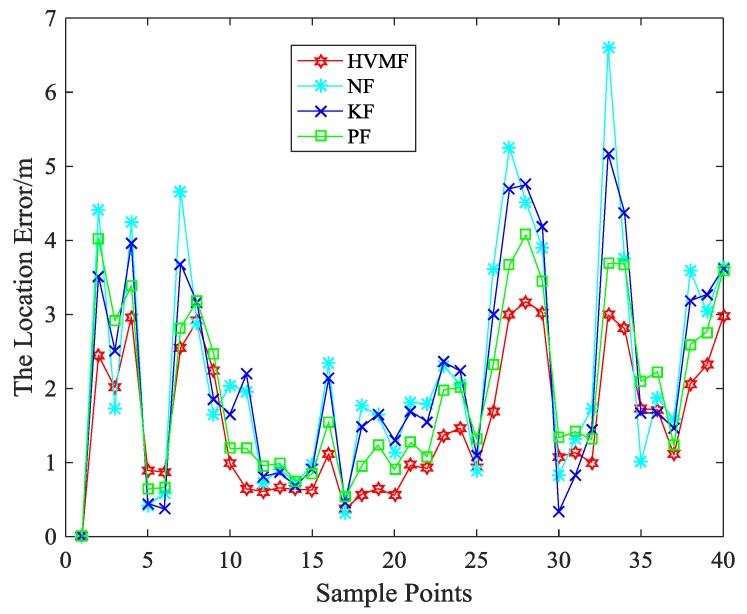
The localization error in each sample point.

The localization error of each algorithm at each sampling site is as shown in Figure 21. The average localization errors of the NF, KF, PF, and HVMF algorithms are 2.3138 m, 2.2119 m, 1.9617 m, and 1.5684 m, respectively. Therefore the proposed method has better performance than the other methods.

**Figure 22:**
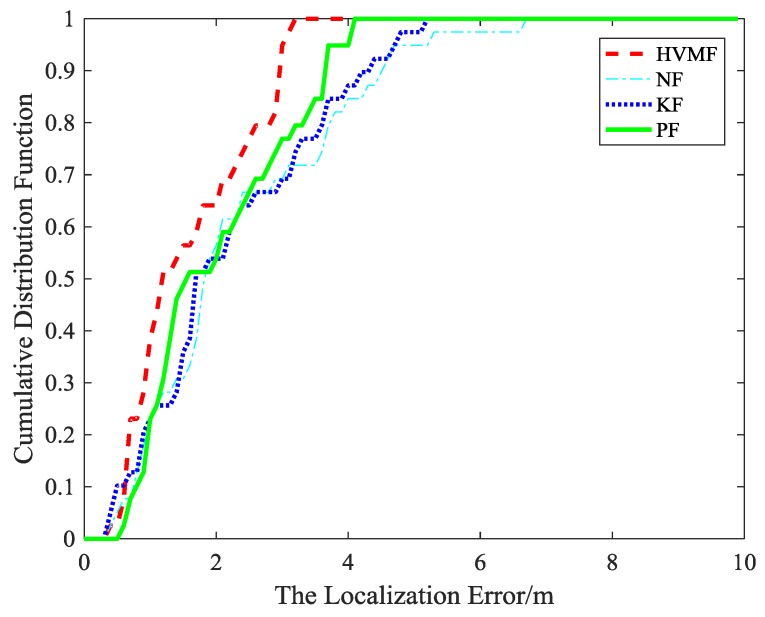
The localization error versus CDF.

The cumulative distribution function of the localization error in the field experiment is shown in Figure 22. It shows that the localization error of the KF, NF, and PF is 5.2 m, 6.8 m, and 4.1 m when the cumulative distribution function is close to 1, while the localization error of the HVMF algorithm is 3.3 m. The 90-percentile of the localization error of the KF, NF, PF, and HVMF algorithms are less than 4.7 m, 4.8 m, 3.8 m, and 3.0 m. It can be seen the HVMF algorithm has the highest localization accuracy.

#### 5.2.2. Computation Time

Table 3 shows the running times of the KF, PF, and HVMF. The three methods are coded using Matlab2014 and tested on a Windows 7 Professional workstation with Intel(R) Core(TM) i5-5200U CPU @ 2.20 GHz and 4.00 GB RAM. Compared with the traditional method, the proposed method has the highest running time, but it is still lower than the total elapsed time for one sampling (0.05 s-the measurement frequency of CSS nodes is set to 20 Hz), meaning that with the computer used here, the algorithm can be applied for online tracking. Therefore, our algorithm remains competitive in terms of computation time.

## 6. Conclusions

This paper proposes a HVMF algorithm based on hierarchical voting for the aim of the localization of mobile nodes in mixed LOS and NLOS environments. The hierarchical voting algorithm proposed by the propagation condition detection and distance correction method does not need to identify the propagation state and has a fast operation speed, and is applicable to TOA, TDOA, RSS, and other measurement methods. At the same time, we use the SRUKF and PF methods to filter the Gaussian noise and non-Gaussian noise, respectively, and use a hierarchical voting algorithm to combine the two sets of data. In addition, we perform convex optimization for the estimated sigma points, which are generated from the mixed measurement values. The points that violated the constraints are projected on the feasible region, and a new set of prediction points are obtained. Moreover, the location estimation method is utilized to get the final position of the mobile node. The simulation results show that the proposed algorithm can efficiently reduce the effect of NLOS errors whether in large measurement noise or small measurement noise. The proposed method could achieve higher localization accuracy when NLOS errors obey Gaussian distribution, uniform distribution, or exponential distribution. The performance of the proposed method is better than no filter method, Kalman filter and PF. And it is robust to the NLOS errors.

For future work, more experiments will be conducted to extend the proposed method to cooperative tracking of multiple mobile nodes. The computational complexity could also be reduced through the optimization of the method. At the same time, we will modify the Hierarchical Voting algorithm and apply it to RSS-based positioning to improve the effectiveness of RSS positioning.

## Figures and Tables

**Table 1:** List of Key Notations.

Notation	Explanation	Notation	Explanation
N	the number of beacon nodes	$Z_{i}$	the position of beacon nodes
$Z_{u}^{t}$	the position of mobile nodes	$d_{k}^{i}$	the true distance between the *i*-th beacon node and the mobile node at time *k*
${\hat{d}}_{k}^{i}$	the measured distance measurement of the *i*-th beacon node at time *k*	$b_{NLOS}$	the NLOS error
$n_{i}$	the measurement noise	$l_{k}^{i}$	the probability of the measurement contains NLOS error
${\tilde{d}}_{k}^{i\left(SRUKF\right)}$	the output of Square Root Unscented Kalman Filter	${\tilde{d}}_{k}^{i\left(PF\right)}$	the output of Particle Filter
${\tilde{d}}_{k}^{i\left(M\right)}$	the mixed measurement value	$s_{k}^{i}$	the state vector measured by the *i*-th beacon node at time *k*
$P_{k}$	the variance of the state vector $s_{k}^{i}$	$F_{k}$	the state transition matrix
$G_{k}$	system process noise input matrix	$\omega_{k}$	process noise
$H_{k}$	observation matrix	$v_{k}$	observation noise
C(m,n)	the location of each voting node	$d_{imn}$	the Euclidean distance between *i*-th beacon node and the C(m,n)
$b_{i}\left(m,n\right)$	the number of votes increased at C(m,n) given by the measurement of the *i*-th beacon node	V(m,n)	voting result matrix
v	the number of the possible initial estimated position of mobile node	$C^{*}$	the initial results set
${\hat{s}}_{k}$	the average state for group $s_{k}^{i}$	$s_{i,k|k}$	the estimated state measured by the *i*-th beacon node at time *k*
$U_{i,k|k}$	the estimated error covariance matrix of the state measured by the *i*-th beacon node at time *k*	$s_{i,k|k-1}$	the estimated state of $s_{i,k-1|k-1}$
$U_{i,k|k-1}$	the estimated matrix of $U_{i,k-1|k-1}$	$s_{i,k|k-1}^{\left(j\right)}$	the estimated sigma points for group $s_{i,k-1|k-1}$
$N_{v}$	the dimension of the state vector	$\omega^{\left(j\right)}$	the weight coefficient for *i*-th sigma points
$z_{i,k|k-1}^{\left(j\right)}$	the estimated distance from $s_{i,k|k-1}$	${\hat{z}}_{i,k|k-1}$	the average distance for group $z_{i,k|k-1}^{\left(j\right)}$
$P_{i,k|k-1}^{s,z}$	the cross-covariance matrix of $z_{i,k|k-1}^{\left(j\right)}$ and $s_{i,k|k-1}^{\left(j\right)}$	$T_{i,k}$	the filter gain matrix
$N_{s}$	the number of the particles we use in PF	$s_{i,k-1}^{n}$	the estimated state of *i*-th particle
$\omega_{i,k-1}^{n}$	the weight coefficient for particles	$d_{k}^{\left(j\right)}$	the estimated sigma points for group ${\tilde{d}}_{k}^{i\left(M\right)}$
$P\left(d_{k}^{\left(j\right)}\right)$	the optimized point for the group $d_{k}^{\left(j\right)}$	${\tilde{d}}_{k}^{i}$	the output of convex optimization

**Table 2:** The default parameter values.

Parameters	Symbol	Default Values
The number of beacon nodes	*N*	8
The standard deviation of measurement noise	$\sigma_{i}$	1
The NLOS error	$N\left(\mu_{NLOS},\sigma_{NLOS}^{2}\right)$	*N*(2,3^2^)

**Table 3:** Running times of each method.

Method Used	Running Times/s
KF	0.0015
PF	0.0045
HVMF	0.0324
